# Diagnostic findings caused by cutting of the iliotibial tract and anterolateral ligament in an ACL intact knee using a standardized and automated clinical knee examination

**DOI:** 10.1007/s00167-017-4499-5

**Published:** 2017-03-17

**Authors:** Timothy Lording, Shaun K. Stinton, Philippe Neyret, Thomas P. Branch

**Affiliations:** 1 Melbourne Orthopaedic Group, Melbourne, VIC Australia; 2 ArthroMetrix LLC, 441 Armour Place NE, Atlanta, GA 30324 USA; 3 Department Orthopedic Surgery, Centre Albert-Trillat, Hôpital de la Croix-Rousse, Lyon, France; 4 University Orthopedics, Decatur, GA USA

**Keywords:** Knee ligaments, Knee laxity, Ligament biomechanics, Anterolateral ligament

## Abstract

**Purpose:**

The purpose of this study was to evaluate the separate contribution of the two definitions of the anterolateral ligament (ALL), the mid-third lateral capsular ligament (MTLCL) and deep capsule-osseous layer of the iliotibial tract (dcITT) in addition to the superficial iliotibial tract (sITT) to the control of tibial motion with respect to the femur during the application of force/torque seen during the three tests of the standard clinical knee examination (AP Lachman test, tibial axial rotation test and varus–valgus stress test).

**Methods:**

Six pelvis-to-toe cadaveric specimens were examined using an automated testing device that carried out the three components of the clinical knee examination. Internal/external rotation torque, anteroposterior load and adduction/abduction torque were applied, while torque/force and positional measurements were recorded. Sequential sectioning of the structures followed the same order for each knee, sITT, dcITT and MTLCL. Testing was repeated after release of each structure.

**Results:**

During the tibial axial rotation test, releasing the sITT caused an increase in internal rotation of 2.6° (1.4–4.1°, *p* < 0.0005), while release of the dcITT increased internal rotation an additional 0.8° (0.4–1.1°, *p* < 0.0015). Changes in secondary motions of the tibia after sITT release demonstrated an increase in anterior translation of 1.2 mm (0.6–2.0 mm, *p* < 0.0005) during internal rotation, while release of the dcITT increased the same motion an additional 0.4 mm (0.2–0.5 mm, *p* < 0.0005). During the AP Lachman test, release of the sITT caused the tibia to move more anteriorly by 0.7 mm (0.4–1.1 mm, *p* < 0.0005) and increased internal rotation by 2.7° (0.9–5.2°, *p* < 0.004). The additional release of the dcITT resulted in more anterior translation by 0.3 mm (0.1–0.4 mm, *p* < 0.002) and internal rotation by 0.9° (0.2–1.7°, *p* < 0.005). During the varus–valgus stress test, release of the sITT permitted 0.9° (0.4–1.4°, *p* < 0.0005) more adduction of the tibia, while the additional release of the dcITT significantly increased adduction by 0.4° (0.2°–0.5°, *p* < 0.001). Release of the MTLCL had a nominal but significant increase in internal rotation, 0.6° (0.1–1.1°, *p* < 0.0068) and external rotation, −0.1° (−0.1° to −0.2°, *p* < 0.0025) during the tibial axial rotation test, anterior translation of 0.2 mm (0.0–0.4 mm, *p* < 0.021) only during the AP Lachman test, and adduction rotation, 0.2° (0.0–0.3°, *p* < 0.034) only during the varus–valgus stress test.

**Conclusion:**

The presence of increased adduction during an automated knee examination provides unique information identifying the release of the sITT, dcITT and the MTLCL in this cadaveric study. While their sequential release caused similar pattern changes in the three components of the automated knee examination, the extent of change due to release of the MTLCL was markedly less than after release of the dcITT which was markedly less than after release of the sITT.

## Introduction

The key to the successful treatment of knee ligament injuries is a correct diagnosis. Without a proper diagnosis, ligament injuries are either missed or unnecessary surgery is performed. Ligament injuries result in biomechanical changes within the knee, which can be identified by changes in laxity during the three tests comprising the standard clinical knee examination (AP Lachman test, varus–valgus stress test & tibial axial rotation test). While the clinical knee examination profile is well understood for the major knee ligaments [e.g. Anterior cruciate ligament (ACL), posterior cruciate ligament (PCL), medial and lateral collateral ligaments (MCL, LCL)], profiles for secondary ligamentous structures are not as well understood.

The anterolateral ligament (ALL) has been recently popularized in the literature [2, 6–8, 12, 13, 16–19, 22–24]. Dodds et al. suggest that it is below the deep capsulosseous layer (dcITT) of the iliotibial tract (ITT) but superficial to the capsule [8], while Claes et al. reported that it is a part of the capsular complex [7]. Kittl et al. chose to consider the dcITT and the underlying anterolateral capsule separately [15], while others considered it all-in-one as the anterolateral capsule (ALC) [11]. For this study, two components of the ITT were examined separately: the superficial ITT (sITT) and the deep capsulosseous layer of the ITT (dcITT). In addition, the medial third lateral capsular ligament (MTLCL) was examined separately as another definition of the ALL below the dcITT and as a part of the anterior capsular complex. Discussions of the biomechanical function of these ‘ligaments’ along with the superficial ITT (sITT) have focused on their role in the control of tibial axial rotation and/or anterior translation while limiting review of the other directions of increased “joint play” [6–8, 14, 22]. Furthermore, no cadaveric biomechanical study has been performed looking at the impact of release of the sITT, dcITT and/or MTLCL in the context of the standard clinical knee examination. The goal of this study was to provide information to aid the surgeon in making the appropriate diagnosis of a sITT, dcITT and/or MTLCL (collectively the anterolateral corner) injury in the ACL intact knee. For this study, it was postulated that if injury to the sITT, dcITT and/or MTLCL could not be identified in the ACL intact knee then the importance of these ligaments would be diminished in the face of an anatomic ACL reconstruction.

The purpose of this study was to use an automated knee examination to evaluate how sITT, dcITT and MTLCL integrity impacts knee biomechanics in the ACL intact knee. The hypothesis was that release of the sITT, dcITT and/or MTLCL would result in significant and identifiable changes in the extent and pattern of motion during an automated knee examination that mimics the three components of a clinical knee examination (AP Lachman test, tibial axial rotation test and the varus–valgus stress test).

## Materials and methods

A cadaveric study was undertaken using a previously described automated system [3–5, 20, 21] that mimics the three components of the clinical knee examination (AP Lachman test, tibial axial rotation test and the varus–valgus stress test). The study consisted of sequential release of the sITT, dcITT and MTLCL followed by evaluation of each knee with the three components of the automated knee examination (Table 1). No IRB approval was required for cadaveric studies at the institution where testing was performed.

**Table 1:** This table outlines the sequence of cutting conditions for this study

Test	ACL	ALL/capsule	sITB	dITB	sITB patella	#Knees
1	U	U	U	U	U	12
2	U	U	U	U	C	12
3	U	U	C	U	C	12
4	U	U	C	C	C	12
5	U	C	C	C	C	6

*U* uncut, *C* cut

Six fresh frozen, pelvis to toe cadaveric specimens (12 knees) were used for the study. Specimens with a body mass index (BMI) over 28 kg/m^2^, with a history of lower limb trauma, previous knee surgery or medical conditions that may have impacted the health of the knee, or aged over 75 years were excluded. Potential specimens were screened with radiographs and magnetic resonance imaging (MRI), and specimens with evidence of arthritis, meniscal or ligament damage were also excluded. The mean specimen age was 55 years (35–71 years).

A standardized, automated and reproducible (STAR) knee examination system consisting of six servomotors combined with six torque sensors was used to perform single-axis tests in three planes: (1) external–internal rotation; (2) anterior–posterior; and (3) varus–valgus rotation. For rotational testing, the limb was rotated by a footplate about an approximate centre of tibial rotation 2.5 cm anterior to the heel. Rotation occurred in one direction until a maximum torque of 5 N m was reached, at which point the servomotor changed direction. One rotational cycle was considered to be from 0 N m torque to full external rotation, to full internal rotation, and back to 0 N m torque. For each test, one pre-conditioning cycle and three data acquisition cycles were performed. A similar method was used for anteroposterior translation and varus–valgus testing. For anteroposterior translation, a lever arm was positioned under the calf just distal to the tibial tuberosity with a strap around the leg, and a peak force of 200 N was used. For varus–valgus testing, rotation occurred at the footplate, to a peak torque of 14 N m. These loads were chosen based upon previous in-vivo biomechanical studies with the desire to fully characterize the toe region of load-deformation curves seen in the knee [4].

Positional data were recorded using an electromagnetic motion analysis system (trakSTAR, Ascension Technology Corporation, Shelburne, VT). Sensors were mounted directly to the femur and tibia with screw fixation. Positional data from the electromagnetic system and torque data from the torque sensors were continuously recorded during testing. The motion data was accurate to within 0.48 mm and 0.3**°** (0.88 mm and 0.48**°–**95% confidence interval) based on root mean square error as reported by the manufacturer.

**Fig. 1:**
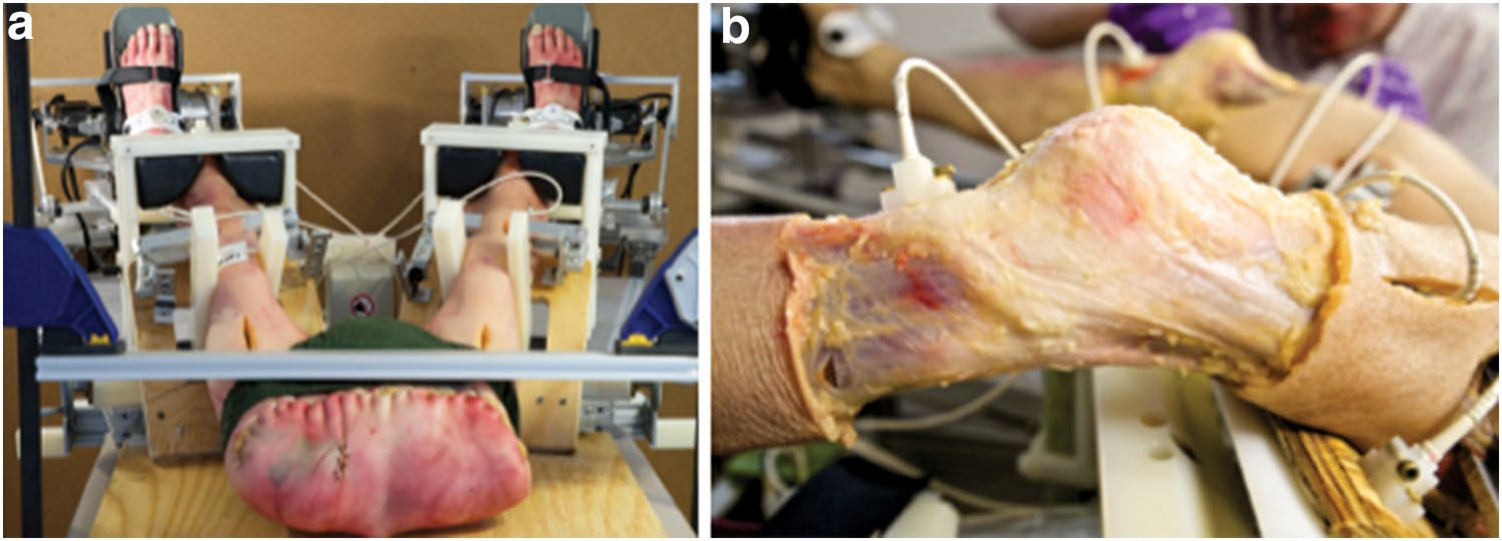
**a** Cadaver setup **b** placement of electromagnetic sensors

Each specimen was stored in a sealed polyethylene bag at −20 °C and thawed at room temperature for at least 24 h prior to testing. The specimen was positioned supine in the automated testing device (Fig. 1) such that the knee joint line was approximately 1 cm distal to the femoral pad. The knees were flexed to 30° and the pelvis was clamped to the examination table. Two incisions were made on both the lateral and medial aspects of the thigh, at approximately the junction of the middle and distal third of the femur. Two metal plates, 3 cm wide, were inserted to clamp the femur. The anterior plate was flat, while the posterior plate was grooved to accommodate the linea aspera. The plates were passed through the fascia lata well anterior and posterior to the borders of the ITT to allow it free movement, and screwed together to control movement of the femur. A diagonal set screw in the anterior plate ensured rotational control. The coronal and rotational position of the limbs was adjusted until the patella faced anteriorly and the feet were then strapped to the footplates. A 3 cm incision was made over the anteromedial surface of each tibia, just medial and distal to the tibial tuberosity. A mount for the electromagnetic tracking sensor was affixed to the tibia using two brass screws to avoid interference. Care was taken to avoid damaging the tendons at the pes anserinus. The femoral sensors were mounted in a similar fashion to the anterolateral femur just distal to the femoral clamps. Each knee was initially held with a patella clamp applied with 133 N force, after which the femoral stabilizing plates were secured to a board to minimize femoral movement during testing. The electromagnetic tracking system was then calibrated and the patella clamps were removed.

A baseline test cycle (Test 1) was performed, and repeated after removal of the skin and subcutaneous tissues from the level of the thigh clamps to approximately 10 cm distal to the knee (Test 2). The sequential cutting phase of the experiment was then carried out. The sITT was dissected from the lateral aspect of the patella and released at Gerdy’s tubercle, taking care not to damage the deeper structures, and a test cycle performed (Test 3). With the sITT detached, the dcITT was identified and released distally from the tibia. A test cycle was performed with both the sITT and dcITT released (Test 4). Finally, release of the meniscotibial ligament was completed from medial to the lateral collateral ligament to the anterior horn of the lateral meniscus securing a complete release of the MTLCL. After release of the capsular ligaments, Test 5 was performed with the sITT, dcITT and MTLCL all cut. Only 6 of the 12 knees were available for statistical comparison in this group.

Throughout each dissection, digital photographs and notes were taken to document the specific anatomic findings for that specimen. Anatomical measurements were not taken.

### Statistical analysis

Matlab (MathWorks, Inc., Natick, MA) and R (R: A language and environment for statistical computing. R Foundation for Statistical Computing, Vienna, Austria. URL http://www.R-project.org/) were used for data analysis. Using the positional data from the electromagnetic sensors, a kinematic path in all three dimensions was established for the tibia with respect to the femur. Combining this three dimensional kinematic data with the load data from the torque sensors, a load-deformation curve was constructed for each test cycle, with rotation/translation on the *x*-axis and torque/force on the *y*-axis. For the purpose of this study, the load-deformation curve was restricted to tracking the major direction of load application. The load-deformation curve was interpolated for a set of 500 points between −5 and 5 N m for external and internal rotation, −200 and 200 N for anterior and posterior translation, or −14 and 14 N m for valgus–varus rotation respectively. Mean curves were then constructed using the average rotation/translation for each of the 500 standardized *y*-value points from the load-deformation curves across all specimens. The slope of the curve represents rotational stiffness, with a steeper curve representing a stiffer or less compliant knee. Point-wise Wilcoxon signed rank testing (non-parametric) was applied across the 500 points. A pre-study power analysis was performed to determine the number of specimens required to detect a difference between groups equal to the accuracy of the measurement system (0.3**°**). Due to the small standard deviation associated with the automated testing system when testing cadaveric specimens in previous studies, a large number of specimens was not required. A sample size of 12 knees provided a power of 0.97 and a sample size of 6 knees provided a power of 0.80.

## Results

In this study, removal of the skin and subcutaneous tissue had no significant impact on knee kinematics during the automated knee examination. Sectioning of the ligament between the lateral patella and the sITT did have a small but significant impact during each examination. These changes in knee kinematics during each of the automated knee examinations are seen in Tables 2 through 4. The mean difference or mean change due to sectioning of each ligament is shown along with the confidence intervals. The total change in motion between the tibia and the femur during each of the examinations is also included.

**Table 2:** The change in tibial axial rotation during the tibial axial rotation test between each condition and its level of statistical significance

Tibial axial rotation test (external/internal rotation)								
	External rotational torque (−5 N m)				Internal rotational torque (5 N m)			
	Mean change	Lower CI	Upper CI	*p* value	Mean change	Lower CI	Upper CI	*p* value
Primary motion (ER/IR)	In degrees, external rotation (−) and internal rotation (+)							
Untouched knee								
sITT off patella+	−0.3	−0.3	−0.2	**0.0005**	0.2	0.1	0.3	**0.001**
sITT off tibia+	0.1	0.1	0.2	**0.001**	2.4	1.3	3.8	**0.0005**
dcITT off tibia+	0.0	−0.2	0.1	N.S	0.8	0.4	1.1	**0.001**
MTLCL*	−0.3	−0.4	−0.2	**0.0313**	0.1	−0.3	0.4	N.S
Total change in ER/IR	−0.5				3.4			
Secondary motion (add/abd)	In degrees, adduction (−) and abduction (+)							
Untouched knee								
sITT off patella+	0.0	−0.1	0.0	N.S	0.0	−0.1	0.1	N.S
sITT off tibia+	−0.1	−0.1	0.0	**0.0015**	−0.4	−0.6	−0.1	**0.0005**
dcITT off tibia+	−0.1	−0.2	0.0	N.S	−0.1	−0.4	0.0	N.S
MTLCL*	0.0	−0.1	0.1	N.S	0.0	−0.1	0.0	N.S
Total change in add/abd	−0.2				−0.5			

Significant *p* values <0.05 are in bold

+12 knees; *6 knees

**Table 3:** This table identifies the change in anterior/posterior translation during the AP Lachman test between each condition and its level of statistical significance

AP Lachman test (AP Translation)								
	Posterior load (−200 N)				Anterior load (200 N)			
	Mean change	Lower CI	Upper CI	*p* value	Mean change	Lower CI	Upper CI	*p* value
Primary motion (post/ant)	In mm, posterior translation (−) and anterior translation (+)							
Untouched knee								
sITT off patella+	−0.1	−0.1	0	N.S	0.1	0.0	0.2	**0.0425**
sITT off tibia+	0.2	0.1	0.4	**0.0093**	0.6	0.3	1.0	**0.0005**
dcITT off tibia+	0.1	−0.1	0.2	N.S	0.3	0.1	0.4	**0.0015**
MTLCL*	0.0	−0.2	0.2	N.S	0.1	−0.2	0.4	N.S
Total change in AP	0.3				1.1			
Secondary motion (ER/IR)	In mm, posterior translation (−) and anterior translation (+)							
Untouched Knee								
sITT off patella+	−0.2	−0.4	0.1	N.S	0.6	0.1	1.1	**0.021**
sITT off tibia+	0.5	−0.2	1.3	N.S	2.1	0.7	4.0	**0.0034**
dcITT off tibia+	−0.2	−0.9	0.4	N.S	0.8	0.2	1.7	**0.0049**
MTLCL*	0.1	−0.7	1.2	N.S	−0.1	−1.0	0.7	N.S
Total change in ER/IR	0.2				3.5			

Significant *p* values <0.05 are in bold

+12 knees; *6 knees

**Table 4:** This table identifies the change in adduction/abduction rotation during the varus–valgus stress test between each condition and its level of statistical significance

Varus–valgus stress test (adduction/abduction rotation)								
	Adduction rotation torque (−14 N m)				Abduction rotation torque (14 N m)			
	Mean change	Lower CI	Upper CI	*p* value	Mean change	Lower CI	Upper CI	*p* value
Primary motion (add/abd)	In degrees, adduction (−) and abduction (+)							
Untouched knee								
sITT off patella+	−0.1	−0.1	0.0	**0.0234**	0.0	0.0	0.0	N.S
sITT off tibia+	−0.8	−1.3	−0.4	**0.0005**	−0.1	−0.2	0.0	N.S
dcITT off tibia+	−0.3	−0.5	−0.2	**0.0010**	0.0	−0.1	0.1	N.S
MTLCL*	−0.1	−0.2	0.0	N.S	0.0	−0.1	0.1	N.S
Total change in ER/IR	−1.3				−0.1			
Secondary motion (ER/IR)	In degrees, external rotation (−) and internal rotation (+)							
Untouched knee								
sITT off patella+	0.1	−0.2	0.2	N.S	0.1	−0.1	0.3	N.S
sITT off tibia+	2.5	1.1	4.3	**0.0005**	0.8	0.4	1.2	**0.0005**
dcITT off tibia+	0.4	0.2	0.8	**0.0049**	0.1	−0.1	0.3	N.S
MTLCL*	0.0	0.1	−0.3	N.S	−0.3	−0.1	−0.6	**0.0313**
Total change in ER/IR	3.0				0.7			

Significant *p* values <0.05 are in bold

+12 knees; * 6 knees

**Fig. 2:**
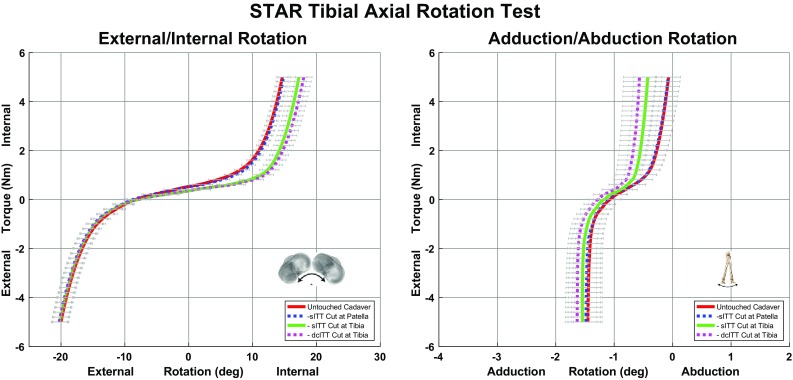
Results of the tibial axial rotation test showing the primary motion of external/internal rotation (*left*) and the secondary motion of abduction/abduction (*right*)

The results for the tibial axial rotation test after sequential cutting of the sITT at the patella, sITT and dcITT are presented in Fig. 2. Load-deformation curves representing motions resulting from the application of an external/internal rotational torque during the test are presented. Changes in extent are visualized by curve displacement and changes in stiffness are visualized by slope differences. All mean changes between curves at the endpoints are listed in Table 2 for the primary direction and most significant secondary direction during the torque application for the tibial axial rotation test. The majority of change occurred after sectioning of the sITT resulting in increased internal rotation and adduction during the application of internal rotation torque. While the dcITT mimicked the sITT, motion was to a lesser extent. The MTLCL had a nominal effect only during external rotation torque.

**Fig. 3:**
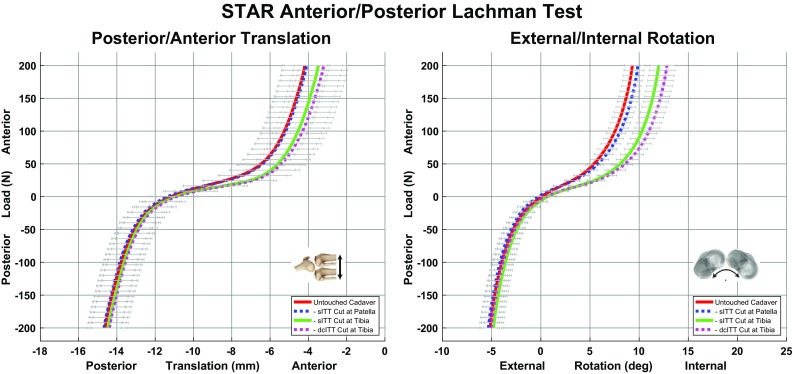
Results of the anterior/posterior Lachman test with the primary motion of posterior/anterior translation (*left*) and the secondary motion of external/internal rotation (*right*)

The results for the AP Lachman test after sequential cutting of the sITT at the patella, sITT and dcITT are presented in Fig. 3. All mean changes between curves at the endpoints are listed in Table 3 for the primary direction and most significant secondary direction during the torque application for the AP Lachman Test. The majority of change occurs after release of the sITT resulting in increased anterior translation and internal rotation during the application of an anterior load. While the dcITT mimicked the sITT, the motion was to a lesser extent. The MTLCL had no significant effect on primary or secondary motions of the tibia with respect to the femur during application of an anterior/posterior load during the automated knee examination.

**Fig. 4:**
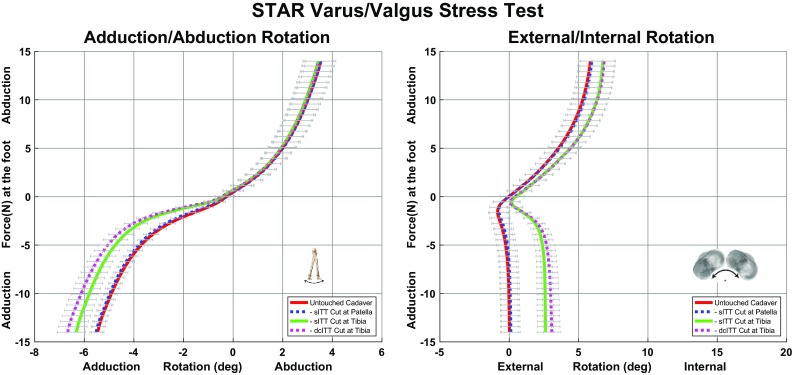
Results of the varus–valgus stress test with the primary motion of adduction/abduction rotation and the secondary motion of external–internal rotation

During the varus–valgus stress test, sequential cutting of the sITT at the patella, sITT and the dcITT caused significant changes to occur (Fig. 4). All mean changes between curves at the endpoints are listed in Table 4 for the primary direction and most significant secondary direction during the torque application for the varus–valgus stress test. The majority of change occurs after sectioning of the sITT resulting in increased adduction rotaton and internal rotation during the application of an adduction torque. While the dcITT mimicked the sITT, the motion was to a lesser extent. The MTLCL had a nominal effect during the application of abduction torque only resulting in a slight increase in external rotation.

In all cadavers, the sITT was found to have an insertion into Gerdy’s tubercle creating a passive structural restraint across the tibiofemoral joint. The structural characteristics of this passive restraint were always found to be robust in nature. The dcITT was identified in all specimens. In some cases, it was a robust and easily identifiable structure, while in others it was flimsy and almost translucent. As noted, there was variation in both the apparent length of this structure and in its insertion point on the tibia, although formal anatomical measurements were not made (see Fig. 1b). Release of the anterolateral meniscotibial ligament effectively released the MTLCL (see Fig. 1c).

## Discussion

The most important finding in this study was that an automated knee examination could identify the sequential release of the sITT and dcITT in the ACL intact knee. This identification could be achieved through the increases in anterior translation and internal rotation during the AP Lachman test, increases in internal rotation and anterior translation during the tibial axial rotation test, and, most importantly, through increases in adduction, internal rotation and anterior translation during the varus–valgus stress test caused by the sequential release of the ligaments. Importantly, the increase in adduction rotation during the varus–valgus stress test may be helpful in uniquely identifying an injury to these structures in the ACL intact knee.

Another important finding of this study was that release of the ALL, considered as the dcITT, had only a small impact on controlling knee motion. Furthermore, increases in motion caused by release of the dcITT mimicked those of the sITB making it clinically indistinguishable from each other. Sectioning of the ‘other’ defined ALL, the MTLCL, had little significant impact on controlling tibial motion with respect to the femur. Results of cutting the anterolateral capsule in the primary motions for each test as well as the most affected secondary motion were reported to provide the clinician with information to improve their clinical knee examination.

The findings from the current study are broadly in line with what is known about the role of the ITT in terms of rotational control. In his article, Glenn Terry combined findings at surgical exposure with findings using the clinical knee examination [22]. It was suggested that damage to the ITT occurred proximally with a focus on injury at the dcITT level. Furthermore, it was found that knees with dcITT damage had more adduction during the clinical knee examination, which was confirmed in the current study. Differentiating between an isolated ACL tear and an ACL with combined anterolateral capsule complex instability is possible through identification of the increased abduction rotation seen after injury to the anterolateral complex. Whether or not this is still valid in an ACL deficient knee will require further study. Gadikota et al., in a robotic study investigating the effect of increasing iliotibial band load, found that internal rotation was significantly reduced between 20° and 30° of knee flexion with an load of 50 N, and from 15° to 30° with a load of 100 N [10]. Fairclough et al., in an anatomical study of the iliotibial band, suggested it consisted of, “a ‘tendinous’ part proximal to the lateral femoral epicondyle and a ‘ligamentous’ part between the epicondyle and Gerdy’s tubercle” [9]. They suggested that this ligamentous portion, from Kaplan’s fibers to Gerdy’s tubercle, may function as an independent static restraint of internal tibial rotation. More recently, Kittl et al. reported the results of a robotic cadaveric study of the role of the sITT and dcITT in internal rotational control [15]. They found the superficial and deep layers of the ITT to be the main restraints to internal rotation and to a simulated pivot shift, with the ALL contributing little to stability. Other studies have suggested that the ALL, considered as the dcITT or the MTLCL, contribute to internal rotation control in varying degrees [1, 9, 15, 16]. All three ligaments, sITT, dcITT and the MTLCL contribute to internal rotational control of the tibia with respect to the femur to some degree. Only the sITT and the dcITT appear to contribute to adduction control of the tibia with respect to the femur. Individual ligament contribution may be less important than the combined impact of injury to all three of the ligaments.

This study is subject to a number of limitations. This cadaveric study involved sequential release of multiple ligamentous structures and is subject to all issues related to cutting order. Furthermore, in vivo injury to the sITT and/or dcITT likely occurs proximally [22], and thus, while our distal release is likely to have the same biomechanical effects, the injury location may be different. While we chose to completely release the anterolateral meniscotibial ligament to section the MTLCL, there may be retained function through the meniscofemoral ligament. In addition only six knees were tested for the MTLCL compared to 12 knees for the sITT or dcITT. All testing was performed at a single knee flexion angle of 30°. Parsons et al. demonstrated a wide variance in the contribution of the dcITT to internal rotation control between specimens at this flexion angle [16]. Our results may have been more consistent across all specimens at a higher or lower knee flexion angle. Due to the high test–retest consistency of the robotic testing device, very small magnitude changes in rotation and displacement may be highly statistically significant, even with a small number of cadaveric specimens. Care should be taken in interpreting the clinical significance of these small changes.

## Conclusion

The presence of increased adduction during an automated knee examination provides unique information identifying the release of the sITT, dcITT and the MTLCL in this cadaveric study. While their sequential release caused similar pattern changes in the three components of the automated knee examination, the extent of change due to release of the MTLCL was markedly less than after release of the dcITT which was markedly less than after release of the sITT.
